# Piwi-like 1 and -2 protein expression levels are prognostic factors for muscle invasive urothelial bladder cancer patients

**DOI:** 10.1038/s41598-018-35637-4

**Published:** 2018-12-06

**Authors:** Markus Eckstein, Rudolf Jung, Katrin Weigelt, Danijel Sikic, Robert Stöhr, Carol Geppert, Abbas Agaimy, Verena Lieb, Arndt Hartmann, Bernd Wullich, Sven Wach, Helge Taubert

**Affiliations:** 1 Institute of Pathology, University Hospital Erlangen, FAU Erlangen-Nürnberg, Germany; 2 Department of Urology and Pediatric Urology, University Hospital Erlangen, FAU Erlangen-Nürnberg, Germany

## Abstract

Piwi-like proteins are essential for stem-cell maintenance and self-renewal in multicellular organisms. We analyzed the expression of Piwi-like 1 and Piwi-like 2 by immunohistochemistry (IHC) in 95 muscle invasive bladder cancer (MIBC) samples using tissue microarray. Application of an immunoreactive score (IRS) revealed 37 and 45 patients who were Piwi-like 1 and -2 positive (IRS > 2). IHC results were correlated with clinico-pathological and survival data. The expression of both proteins was positively correlated with each other, lymph node metastasis and expression of CK20 and GATA 3. A negative correlation for both proteins was detected for disease-specific survival (DSS), recurrence, Ki67/MIB1 proliferation index, and CK5 expression. Detection of Piwi-like 1 protein positivity was associated with poor DSS (P = 0.019; log rank test, Kaplan-Meier analysis), and in multivariate Cox’s analysis (adjusted to tumor stage and tumor grade), it was an independent prognostic factor for DSS (RR = 2.16; P = 0.011). Piwi-like 2 positivity was associated with DSS (P = 0.008) and recurrence-free survival (RFS; P = 0.040), and in multivariate Cox’s analysis, Piwi-like 2 positivity was an independent prognostic factor for DSS (RR = 2.46; P = 0.004) and RFS (RR = 3.0; P = 0.003). Most interestingly, in the basal type patient subgroup (CK5+/GATA3−), Piwi-like 2 positivity was associated with poorer DSS, OS and RFS (P < 0.001, P = 0.004 and P = 0.05; log rank test). In multivariate analysis, Piwi-like 2 positivity was an independent prognostic factor for DSS (RR = 12.70; P = 0.001), OS (RR = 6.62;  = 0.008) and RFS (RR=13.0; P = 0.040). In summary, Piwi-like 1 and -2 positivity are associated with clinico-pathological factors and survival. Both Piwi-like proteins are suggested as biomarkers for MIBC patients.

## Introduction

Bladder cancer (BCa) is the ninth most commonly diagnosed cancer and the 13th leading cause of cancer-related death worldwide^1^. Clinical management of BCa^2,3^, and the etiology and diagnostic, prognostic or predictive biomarkers for BCa have been described extensively^4,5^. While there are treatment options available for both superficial and invasive BCa, metastatic disease still presents a serious clinical problem with limited therapeutic options. Remarkably, similar to breast cancer, BCa can be subdivided in basal and luminal subtypes which harbor prognostic and predictive relevance (e.g. improved neoadjuvant chemotherapy responsiveness)^6–9^. Recently, promising immunotherapeutical PD-1/PD-L1 and/or CTLA4 emerged for the treatment of metastasized BCa^10,11^. However, there is still an urgent need to identify additional useful biomarkers in BCa.

Piwi-like genes belong to the Argonaute gene family, and they are essential for stem cell maintenance and self-renewal in multicellular organisms ranging from plants to humans^12,13^. Piwi-like proteins catalyze an amplification loop (ping-pong cycle) of small RNAs (piRNAs). Both piRNAs and Piwi-like proteins function as a Piwi-ribonucleoprotein complex for transposon repression through target degradation and epigenetic silencing^14,15^. In addition to their expression in the germ-line, an increased (re)expression in different tumors has been described, especially for Piwi-like 1 and Piwi-like 2^16–19^. Silencing of Piwi-like 1 by siRNA suppressed BCL2 and cyclin D1 expression and inhibited cell proliferation by promoting apoptosis in glioma cells^20^. In addition, Cao *et al*. showed that Piwi-like 1 affects the cell cycle by decreasing the expression of transforming growth factor-β receptors (TGFRI/II), and increasing the expression of cyclin-dependent kinases (CDK) 4, CDK6 and CDK8 on the RNA and the protein level in breast cancer cells^21^. An association of Piwi-like 1 (Hiwi) with global DNA methylation and silencing of cyclin-dependent kinase inhibitor (CDKI) has been reported in Hiwi expressing MSCs^22^. In line with these findings, Piwi-like 1 overexpression promoted cell proliferation and induced global DNA methylation in colon cancer cell lines^23^. Silencing of Piwi-like 2 by siRNA suppressed Stat3 and Bclxl expression and induced apoptosis. Therefore, Lee and colleagues suggested that Piwi-like 2 functions as an oncogene by inhibiting apoptosis and promoting proliferation via the STAT3/BCLXL signaling pathway^24^. Piwi-like 2 takes part in chromatin modification by histone H3 acetylation and affects DNA damage repair^25^. The stem cell protein Piwi-like 2 modulates chromatin modifications during cisplatin treatment^26^.

Urothelial cancer of the bladder has been studied on the RNA level for Piwi-like genes^27^. They found that Piwi-like 2 is not expressed in either human normal urothelial cells or bladder cancer cell lines and tissues. Previously, we showed that Piwi-like 2 expression was correlated with disease-specific and progression-free survival of chemotherapy-treated bladder cancer patients^28^. In this study, we analyzed the tumors of 95 MIBC patients for their protein expression of Piwi-like 1 and Piwi-like 2 and associated their expression with clinico-pathological and survival data. Most remarkably, levels of Piwi-like 2 expression could be used to separate a subgroup of MIBC, i.e., the basal type (CK5+/CK20−), into a group possessing better OS, DSS and RFS with Piwi-like 2-negative staining and a group having worse OS, DSS and RFS with Piwi-like 2-positive staining.

## Results

### Piwi-like 1/-2 expression and correlation with clinico-pathological parameters and expression of selected proteins

We studied a cohort of 95 MIBC for their Piwi-like 1 and Piwi-like 2 protein expression by immunohistochemistry (IHC). The clinico-pathological data of the MIBC patients are summarized in Table 1. Piwi-like 1/-2 protein expression was detected in the cytoplasm and assessed in an IRS score.

**Table 1:** Clinico-pathological data for MIBC patients.

Clinico-pathological parameters	Patients^a^
**Total**	95
**Morphology**	
Urothelial carcinoma	93
• Squamous	23
• Sarcomatoid	9
• MPUC	7
• PUC	2
• Other rare subtypes	11
Pure neuroendocrine	1
Pure adenocarcinoma	1
**Gender**	
females	26
males	69
**Age (years)**	
range	41.0–88.0
mean	69.7
median	71.0
**Tumor stage**	
pT2	23
pT3	52
pT4	20
**Tumor stage grouped**	
pT2	23
pT3 + pT4	72
**Tumor grade 1973**	
G2	5
G3	90
**Tumor grade 2016**	
high grade	95
**Lymph node metastasis**	
N0	58
N1/2	29
unknown	8
**Adjuvant chemotherapy**	
yes	27
no	68
**Survival/observation time (months)**	
range	0.8–135.7
mean	39.1
median	25.4
**Overall survival (OS)**	
alive	23
dead	72
**Disease-specific survival (DSS)**	
alive	49
dead	46
**Recurrence-free survival time (months)**	
range	0.8–135.7
mean	38.3
median	20.8
**Recurrence-free survival (RFS)**	
without recurrence	43
with recurrence	38
unknown	14

^a^None of the patients received radiotherapy, only one patient was previously treated with BCG therapy.

**Figure 1:**
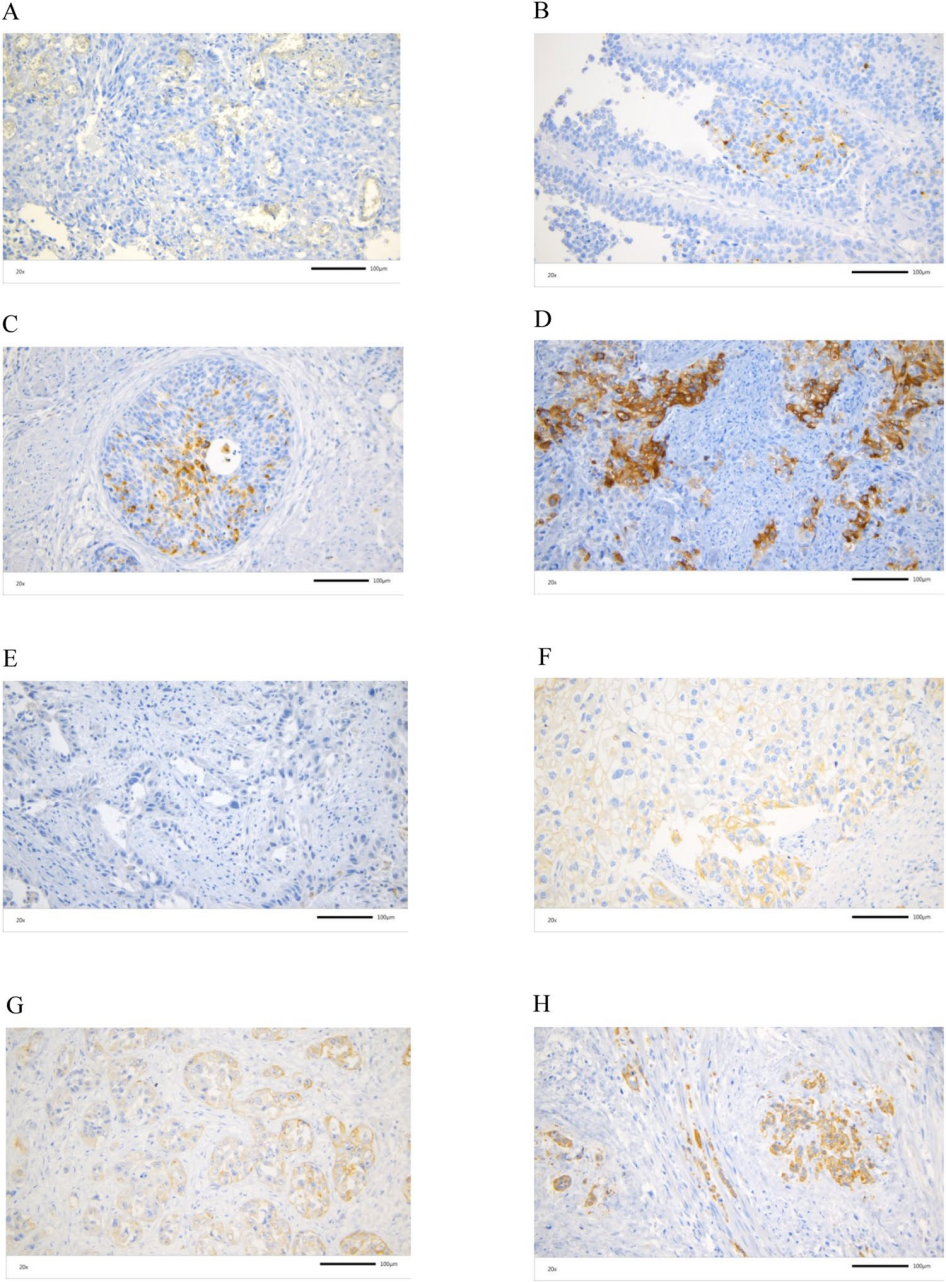
IHC Detection for Piwi-like 1 and Piwi-like 2. Piwi-like 1 staining with IRS = 0 (**A**), IRS = 2 [(intensity 2; percentage <10%] (**B**), IRS = 4 [intensity 2, percentage 20%] (**C**) and IRS = 9 [intensity 3, percentage 75%] (**D**) and Piwi-like 2 staining with IRS = 0 (**E**), IRS = 2 [intensity 2; percentage <10%] (**F**), IRS = 4 [intensity 2, percentage 20%] (**G**) and IRS = 9 [intensity 3, percentage 80%] (H). All photos are at a magnification of ×200 and the scale bar represents 100 µm.

We detected 58 cases (61.1%) with negative Piwi-like 1 staining (IRS ≤ 2) and 37 cases (38.9%) with positive Piwi-like 1 staining (IRS > 2) (Suppl. Table). In addition, there were 50 cases (52.6%) with negative Piwi-like 2 staining (IRS ≤ 2) and 45 cases (47.4%) with positive Piwi-like 2 staining (IRS > 2). Piwi-like 1/-2 protein expression detected by IHC is shown exemplary in Fig. 1.

Next, we tested whether Piwi-like 1 staining was associated with clinico-pathological parameters by correlation tests (Spearman’s bivariate correlation test). There was no association of the Piwi-like 1 IRS with age, gender or tumor size. A significant positive association was found for the Piwi-like 1 IRS with lymph node metastasis (r_s_ = 0.224; P = 0.029), Piwi-like 2 staining (r_s_ = 0.730; P < 0.001), CK20 staining (r_s_ = 0.406; P < 0.001), or GATA 3 staining (r_s_ = 0.363; P < 0.001). A negative correlation with disease-specific survival (r_s_ = −0.281; P = 0.006), time to recurrence (r_s_ = −0.225; P = 0.044), the MIB1 staining (r_s_ = −0.276; P = 0.007), and CK5 staining (r_s_ = −0.385; P < 0.001) was detected.

There was also no association of the Piwi-like 2 IRS with age, gender or tumor size. A significant positive association of the Piwi-like 2 IRS with lymph node metastasis (r_s_ = 0.308; P = 0.002), Piwi-like 1 staining (r_s_ = 0.730; P < 0.001), CK20 staining (r_s_ = 0.464; P < 0.001), and GATA 3 staining (r_s_ = 0.499; P < 0.001) was detected. A negative correlation with the disease-specific survival (r_s_ = −0.311; P = 0.002), time to recurrence (r_s_ = −0.344; P = 0.002), the MIB1 staining (r_s_ = −0.238; P = 0.020), and CK5 staining (r_s_ = −0.322; P = 0.001) was identified.

### Association of Piwi-like 1/-2 protein expression and survival

**Figure 2:**
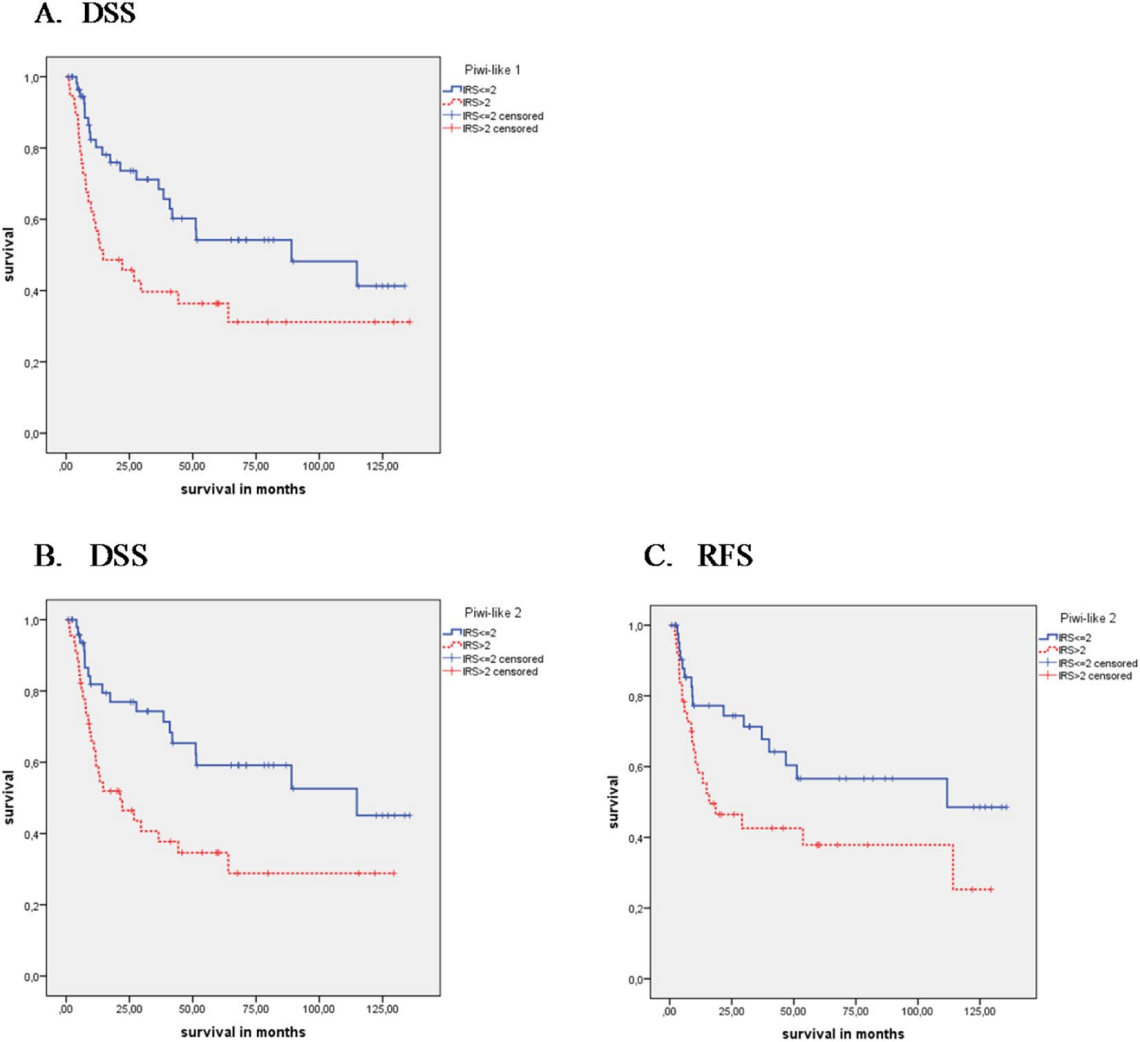
Kaplan-Meier analyses: Association of Piwi-like 1/-2 staining with prognosis in all MIBC patients Piwi-like 1 protein expression was correlated with (**A**) DSS (P = 0.019; log rank test, Kaplan-Meier analysis) Piwi-like 2 protein expression was correlated with (**B**) DSS (P = 0.008) and (**C**) RFS (P = 0.040; both log rank tests, Kaplan-Meier analyses).

There was no association of Piwi-like 1 staining with OS (P = 0.486) or RFS (P = 0.150) but a significant association with DSS (P = 0.019) could be observed by Kaplan-Meier analysis (Table 2; Fig. 2). Here, the mean disease-specific survival time was 53.2 months for Piwi-like 1-positive patients vs. 79.3 months for Piwi-like 1-negative patients. Univariate Cox’s regression analysis revealed that Piwi-like 1 positivity was associated with a 1.98-fold increased risk of tumor-specific death (P = 0.021; Table 3). Multivariate Cox’s regression analysis (adjusted for tumor grade and tumor stage) revealed that Piwi-like 1 staining was an independent predictor of DSS (relative risk (RR) = 2.16; P = 0.011; Table 3).

**Table 2:** Kaplan-Meier analysis: Association of Piwi-like 1/-2 staining with OS, DSS or RFS.

			Kaplan-Meier analysis					
**Piwi-like 1**	**N**	**OS**		**DSS**		**N**	**RFS**	
**IRS > 2 vs. IRS ≤ 2**								
		**months**	**P**	**months**	**P**		**months**	**P**
all patients	95		n.s.	53.2 vs. 79.3	0.019	81		n.s.
Tumor stage 3 + 4	72		n.s.	44.8 vs. 74.9	0.011	62		n.s.
Ki67 <= 30%	71		n.s.	47.5 vs. 73.2	0.030	61		n.s.
CK5+/GATA3−	31		n.s.		n.s.	27		n.s.
CK5−/GATA3+	23	24.7 vs. 65.7	0.049	27.4 vs. 90.1	0.014	19		n.s.
Squamous subtype	23	8.5 vs. 69.9	0.003	8.5 vs. 91.8	0.003		8.9 vs. 93.0	0.003
**Piwi-like 2**	**N**	**OS**		**DSS**		**N**	**RFS**	
**IRS > 2 vs. IRS ≤ 2**								
		**months**	**P**	**months**	**P**		**months**	**P**
all patients	95		n.s.	50.0 vs. 85.2	0.008	81	55.2 vs. 84.2	0.040
Tumor stage 3 + 4	72		n.s.	45.7 vs. 82.2.	0.017	62	47.7 vs. 80.7	0.046
Ki67 <= 30%	71		n.s.	45.1 vs. 80.8	0.013	61		n.s.
CK5+/GATA3−	31	5.9 vs. 63.9	0.004	5.9 vs. 82.7	<0.001	27	8.7 vs. 82.2	0.05
CK5−/GATA3+	23		n.s.		n.s.	19		n.s.
Squamous subtype	23	8.1 vs. 78.4	<0.001	8.1 vs. 103.9	<0.001		8.9 vs. 93.0	0.003

**Table 3:** Univariate and multivariate Cox’s regression analyses: Association of Piwi-like 1/-2 staining with OS, DSS or RFS.

Univariate Cox’s Regression Analysis								
**Piwi-like 1**	**N**	**OS**		**DSS**		**N**	**RFS**	
**IRS > 2 vs. IRS ≤ 2**								
		**RR**	**P**	**RR**	**P**		**RR**	**P**
all patients	95		n.s.	1.9	0.021	81		n.s.
Tumor stage 3 + 4	72		n.s.	2.1	0.013	62		n.s.
Ki67 <= 20%	71		n.s.	1.9	0.033	61		n.s.
CK5+/GATA3−	31		n.s.		n.s.	27		n.s.
CK5−GATA3+	23		n.s.	5.1	0.028	19		n.s.
Squamous subtype	23	5.6	0.007	7.1	0.011		9.7	0.015
**Univariate Cox’s Regression Analysis**								
**Piwi-like 2**	**N**	**OS**		**DSS**		**N**	**RFS**	
**IRS > 2 vs. IRS ≤ 2**								
		**RR**	**P**	**RR**	**P**		**RR**	**P**
all patients	95		n.s.	2.2	0.009	81	1.9	0.043
Tumor stage 3 + 4	72		n.s.	2.2	0.020	62		n.s.
Ki67 <= 30%	71		n.s.	2.2	0.016	61		n.s.
CK5+/GATA3−	31	5.83	0.011	10.6	0.001	28	6.97	(0.093)
CK5−/GATA3+	23		n.s.		n.s.	19		n.s.
Squamous subtype	23	10.4	0.001	23.9	0.004		9.7	0.015
**Multivariate Cox’s Regression Analysis**								
**Piwi-like 1**	**N**	**OS**		**DSS**		**N**	**RFS**	
**IRS > 2 vs. IRS ≤ 2**								
		**RR**	**P**	**RR**	**P**		**RR**	**P**
all patients	95		n.s.	2.16	0.011	81		n.s.
Tumor stage 3 + 4	72		n.s.	2.10	0.017	62		n.s.
Ki67 <= 30%	71		n.s.	2.09	0.024	61		n.s.
CK5+/GATA−	31		n.s.		n.s.	28		n.s.
CK5-GATA3+	23		n.s.	4.7	(0.068)	19		n.s.
Squamous subtype	23	4.7	0.020	5.1	0.037		9.4	0.028
**Multivariate Cox’s Regression Analysis**								
**Piwi-like 2**	**N**	**OS**		**DSS**		**N**	**RFS**	
**IRS > 2 vs. IRS ≤ 2**								
		**RR**	**P**	**RR**	**P**		**RR**	**P**
all patients	95		n.s.	2.46	0.004	81	3.00	0.003
Tumor stage 3 + 4	72		n.s.	2.20	0.021	62		n.s.
Ki67 <= 20%	71		n.s.	2.54	0.008	61		n.s.
CK5+/GATA−	31	6.62	0.008	12.70	0.001	28	13.0	0.040
CK5-/GATA3+	23		n.s.			19		n.s.
Squamous subtype	23	8.9	0.004	16.9	0.011		9.4	0.028

Concerning Piwi-like 2 staining, patients with positive staining in their tumors showed a shorter OS and DSS than patients with negative staining. The mean survival time for Piwi-like 2-positive patients was 39.7 months vs. 79.1 months, but for Piwi-like 2-negative patients, there was only a non-significant trend (P = 0.057). In DSS, patients with Piwi-like 2-positive tumors had a mean survival of 50.0 months vs. 85.2 months for patients with negative Piwi-like 2 staining (P = 0.008; Table 2; Fig. 2). A univariate Cox’s regression analysis revealed that Piwi-like 2-positive staining was associated with a 1.58-fold risk of death, but this was not significant (P = 0.059), and there was a 2.21-fold increased risk for tumor-specific death (P = 0.009; Table 3). In a multivariate Cox’s regression analysis (adjusted for tumor grade and tumor stage), positive Piwi-like 2 staining was associated with OS (RR = 1.60; P = 0.056) but this was not significant. However, Piwi-like 2 positivity appeared as an independent prognostic factor for DSS in multivariate analysis (RR = 2.46; P = 0.004; Table 3).

In addition, for 81 patients, data for recurrence free survival (RFS) were available. There was no association between Piwi-like 1 positivity and RFS. However, compared with Piwi-like 2 negativity, Piwi-like 2 positivity was associated with a shorter RFS (55.2 months vs. 84.2 months; P = 0.040; Table 2). Univariate Cox’s regression analysis showed that Piwi-like 2 positivity was associated with a 1.95-fold increased risk for recurrence (P = 0.043; Table 3). Multivariate Cox’s regression analysis (adjusted for tumor grade and tumor stage) revealed that Piwi-like 2 positivity was an independent factor for RFS (RR = 3.0; P = 0.003; Table 3).

### Association of Piwi-like 1/-2 protein expression and survival stratified to clinico-pathological parameters

#### Piwi-like 1/-2 protein expression and survival in the pT2 and pT3 + 4 groups

Next, we were interested to see if there were differences in prognosis between the two tumor stage groups (pT2 vs. pT3 + 4) that were associated with Piwi-like 1 or -2 staining. There was no difference in OS and RFS in both tumor stage groups for Piwi-like 1 staining. However, patients in the pT3 + 4 group showed significant differences in DSS with a mean survival of 44.8 months for Piwi-like 1-positive patients compared with 74.9 months for Piwi-like 1-negative patients (P = 0.011; Table 2). Univariate and multivariate Cox’s regression analysis (adjusted for the tumor grade) showed in both analyses a 2.1-fold (P = 0.013 and P = 0.017; Table 3) higher risk of tumor-related death in the Piwi-like 1-positive patients than in the negative ones.

Piwi-like 2 staining could separate the pT3 + 4 group patients with different DSS and RFS but not OS. Patients with Piwi-like 2-positive tumors had an average tumor-specific survival of 45.7 months, whereas those with Piwi-like-negative tumors had an average of 82.2 months (P = 0.017; Table 2). Univariate and multivariate Cox’s regression analysis (adjusted by tumor grade) revealed in both analyses a 2.2-fold (P = 0.020 and P = 0.021) increased risk for tumor-specific death (Table 3). Compared with Piwi-like 2 negativity, Piwi-like 2 positivity was also associated with a shorter RFS (47.7 months vs. 80.7 months; P = 0.046; Table 2). Univariate and multivariate Cox’s regression analysis showed that Piwi-like 2 positivity was associated with a 2.0 and a 1.9-fold increased risk for recurrence but this was not significant (P = 0.051 and P = 0.058; Table 3).

#### Piwi-like 1/-2 protein expression and survival in partially squamous and non-squamous differentiated BCa

Since bladder cancers with squamous histological features are considered distinct from conventional urothelial cancers^6^, we examined the two subgroups squamous (N = 23) and non-squamous BCa (N = 72) separately for an association of Piwi-like1 or -2 staining with prognosis. We detected different associations between Piwi-like 1 or -2 staining and prognosis in the squamous differentiated subtype but not in the non-squamous differentiated subtype.

**Figure 3:**
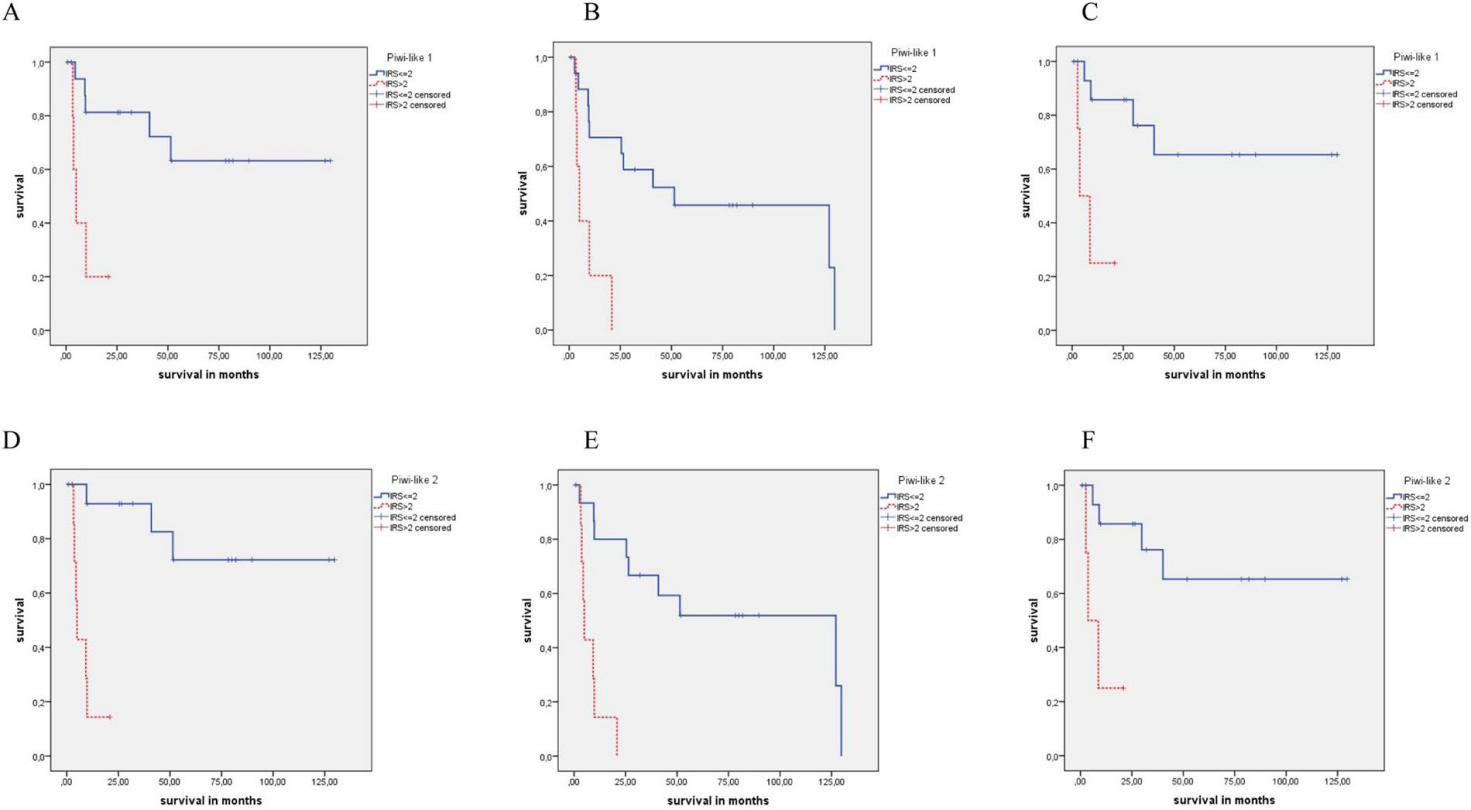
Kaplan-Meier analyses: Association of Piwi-like 1/-2 staining with prognosis in squamous differentiated BCa patients. Piwi-like 1 protein expression was associated with (**A**) DSS, (**B**) OS and (**C**) RFS (all P = 0.003); and Piwi-like 2 protein expression was associated with (**D**) DSS, (**E**) OS (both P < 0.001) and (**F**) RFS (P = 0.003).

In detail, positive Piwi-like 1 staining was significantly associated with OS, DSS and RFS (all P = 0.003). Patients with Piwi-like 1-positive tumors had a mean of overall survival of 8.5 months, disease-specific survival of 8.5 months and recurrence free survival of 8.9 months whereas those with Piwi-like 1-negative tumors survived on average 69.9 months, disease-specific 91.8 months and recurrence free 93.0 months (Table 2; Fig. 3). Univariate Cox’s regression analysis showed an 5.6-fold increased risk for death, a 7.1-fold increased risk for disease-specific death and a 9.7-fold increased risk for recurrence in the Piwi-like 1-positive group compared to the negative group (P = 0.007; P = 0.011 and P = 0.015; Table 3). In multivariate analysis (adjusted to tumor grade and tumor stage) the Piwi-like 1-positive group had a 4.7-fold increased risk of death, a 5.1-fold increased risk of disease-specific death and a 9.4-fold increased risk for recurrence compared to the Piwi-like 1-negative group (P = 0.020; P = 0.037 and P = 0.028; Table 3), i.e., Piwi-like 1 staining was an independent prognostic factor in the squamous differentiated subtype of BCa.

In addition, positive Piwi-like 2 staining was significantly associated with OS, DSS (both P < 0.001) and RFS (P = 0.003; Fig. 3). Patients with Piwi-like 2-positive tumors had a mean of overall survival of 8.1 months, disease-specific survival of 8.1 months and recurrence free survival of 8.9 months whereas those with Piwi-like 2-negative tumors survived on average 78.4 months, disease-specific 103.9 months and recurrence free 93.0 months (Table 2; Fig. 3). Univariate Cox’s regression analysis showed an 10.4-fold increased risk for death, a 23.9-fold increased risk for disease-specific death and a 9.7-fold increased risk for recurrence in the Piwi-like 2-positive group compared to the negative group (P = 0.001; P = 0.004 and P = 0.015; Table 3). In multivariate analysis (adjusted to tumor grade and tumor stage), the Piwi-like 2-positive group had a 8.9-fold increased risk of death, a 16.9-fold increased risk of disease-specific death and a 9.4-fold increased risk for recurrence compared to the Piwi-like 2-negative group (P = 0.004; P = 0.011 and P = 0.028; Table 3), i.e., also Piwi-like 2 staining was an independent prognostic factor in the squamous differentiated subtype of BCa.

#### Piwi-like 1/-2 protein expression and survival in the Ki67 (≤30% vs. >30%) groups

Ki67 staining is associated with prognosis e.g. in breast cancer patients^29^. We separated our patients into two groups by an optimized Ki67 cut-off value of 30%, i.e., a group with ≤30% Ki67 staining (N = 71) and a group with >30% Ki67 staining (N = 24). We evaluated whether we could see differences between the two Ki67 staining groups in prognosis that were associated with Piwi-like 1 or -2 staining. We saw differences in the ≤30% Ki67 group only, and this was for DSS related to the Piwi-like 1 staining. Patients with Piwi-like 1-positive tumors had a mean of tumor-specific survival of 47.5 months, whereas those with Piwi-like 1-negative tumors survived on average 73.2 months (P = 0.030; Table 2). Univariate Cox’s regression analysis showed an 1.9-fold increased risk for disease-specific death in the Piwi-like 1-positive group compared to the negative group (P = 0.033; Table 3). In multivariate analysis (adjusted to tumor grade and tumor stage), the Piwi-like 1-positive group had a 2.1-fold increased risk of disease-specific death compared to the Piwi-like 1-negative group (P = 0.024; Table 3), i.e., Piwi-like 1 staining was an independent prognostic factor.

Again, we detected differences in the ≤30% Ki67 group only for DSS but not for OS and RFS related to Piwi-like 2 staining. Patients with Piwi-like 2 positivity had an average tumor-specific survival of 45.1 months, and those with Piwi-like 2 negativity had an average of 80.8 months (P = 0.013; Table 2). Univariate and multivariate Cox’s regression analysis (adjusted to tumor grade and tumor stage) showed a 2.2-fold and a 2.5-fold increased risk for tumor-associated death in the Piwi-like 2-positive group (P = 0.016 and P = 0.008; Table 3), Piwi-like 2 staining was again an independent factor for DSS in multivariate analysis.

### Association of Piwi-like 1/-2 protein expression and survival stratified to molecular-pathological parameters

#### Piwi-like 1/-2 protein expression in the basal or luminal types of BCa

Different molecular classification systems^6,7^ describe a basal type characterized mainly by CK5 positivity and GATA3 negativity and a luminal type distinguished by CK5 negativity and GATA3 positivity. Although this classification is mainly based on mRNA expression of the markers, protein expression was determined and applied for group determination as well^6,7^. In our patient group, we could determine protein expression of CK5 and GATA3 for 89 patients. Out of these, 31 patients (basal type) were CK5+/GATA3− and 23 patients (luminal type) were GATA3+/CK5−. In addition, 12 patients were negative (CK5−/GATA3−) and 23 patients were positive (CK5+/GATA3+) for both markers. We describe the association of prognosis with the expression of Piwi-like 1 and Piwi-like 2 in the two groups with basal type or luminal type.

#### Piwi-like 1/-2 protein expression and survival in the basal type of BCa

First, we tested whether Piwi-like 1/-2 staining was associated with OS, DSS or RFS in the basal type (CK5+/GATA3− group).

Piwi-like 1 positivity was not significantly associated with OS, DSS and RFS.

**Figure 4:**
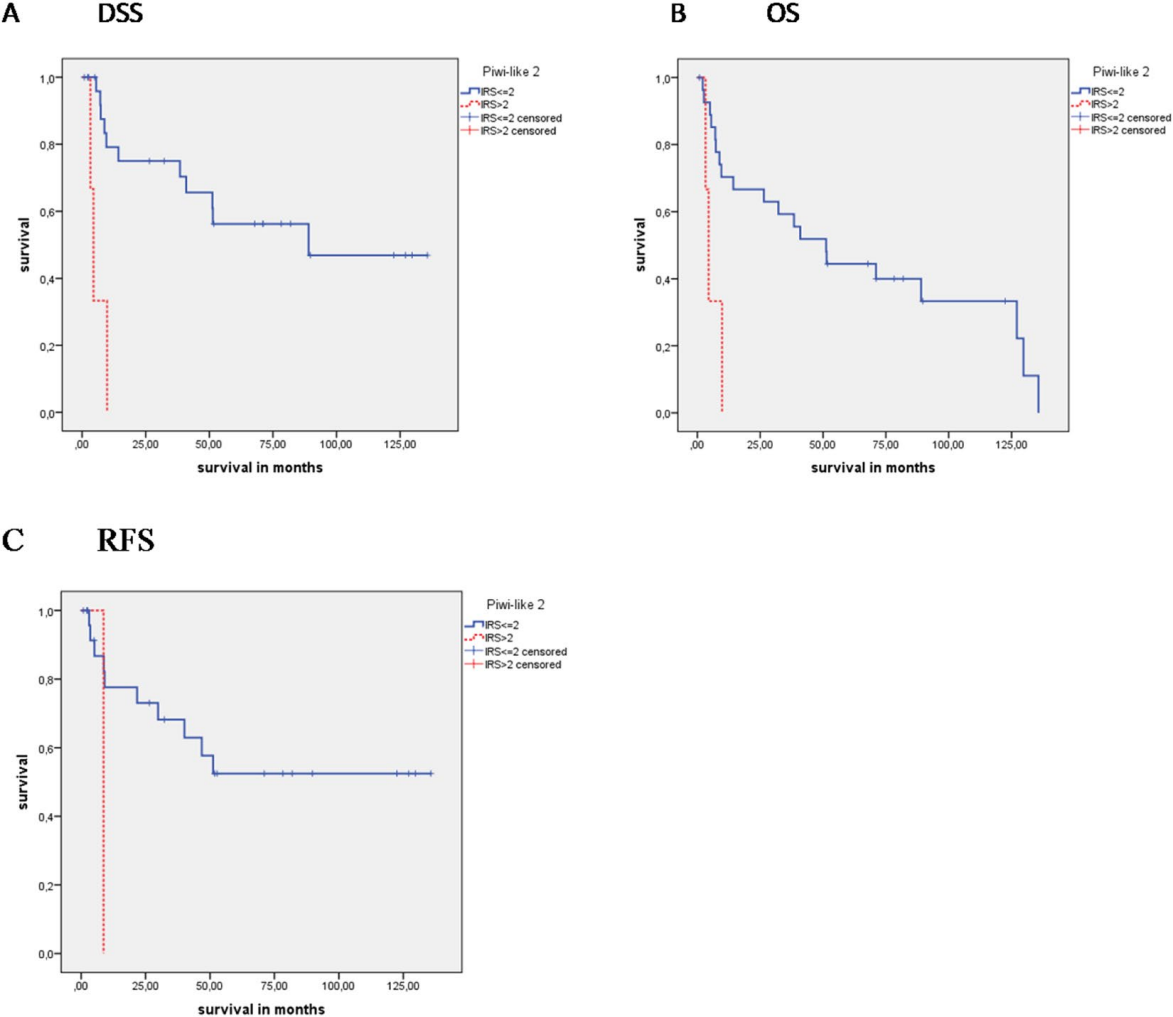
Kaplan-Meier analyses: Association of Piwi-like 1/-2 staining with prognosis in CK5+/GATA3− patients **(**basal subtype) Piwi-like 2 protein expression was associated with (**A**) DSS (P < 0.001), (**B**) OS (P = 0.004) and (**C**) RFS (P = 0.05; all log rank tests, Kaplan-Meier analyses).

Piwi-like 2 positivity was significantly associated with OS and DSS in the CK5+/GATA3− group. Patients with Piwi-like 2 positivity had both an overall and tumor-specific survival of 5.9 months, whereas those with negative Piwi-like 2 staining had an overall survival of 63.9 months with a tumor-specific survival of 82.7 months (P = 0.004 and P < 0.001; Table 2; Fig. 4).

Univariate Cox’s regression analysis showed that, compared with Piwi-like 2 negativity, Piwi-like 2 positivity had a 5.83-fold risk for death and a 10.57-fold risk for tumor-specific death (P = 0.011 and P = 0.001; Table 3). Multivariate Cox’s regression analysis (adjusted for tumor grade and tumor stage) revealed a 6.62-fold risk of death and a 12.70-fold risk for tumor-specific death (P = 0.008 and P = 0.001; Table 3).

In addition, Piwi-like 2 positivity was significantly associated with RFS in the CK5+/GATA3− (N = 27). Comparable to OS and DSS, CK5+/GATA3− patients with Piwi-like 2 positivity had a RFS of only 8.7 months, whereas those with Piwi-like 2 negativity had a RFS of 82.2 months (P = 0.05; Table 2; Fig. 4). Univariate and multivariate Cox’s regression analysis revealed that Piwi-like 2 positivity was associated with a 6.97 and 13.0-fold increased risk of recurrence but this was only significant in the multivariate analysis (P = 0.093 and P = 0.04; Table 3).

Piwi-like 2 positivity appeared to be associated with poorer DSS, OS and RFS in the MIBC patients of the basal type (CK5+/GATA3−).

#### Piwi-like 1/-2 protein expression and survival in the luminal type of BCa

**Figure 5:**
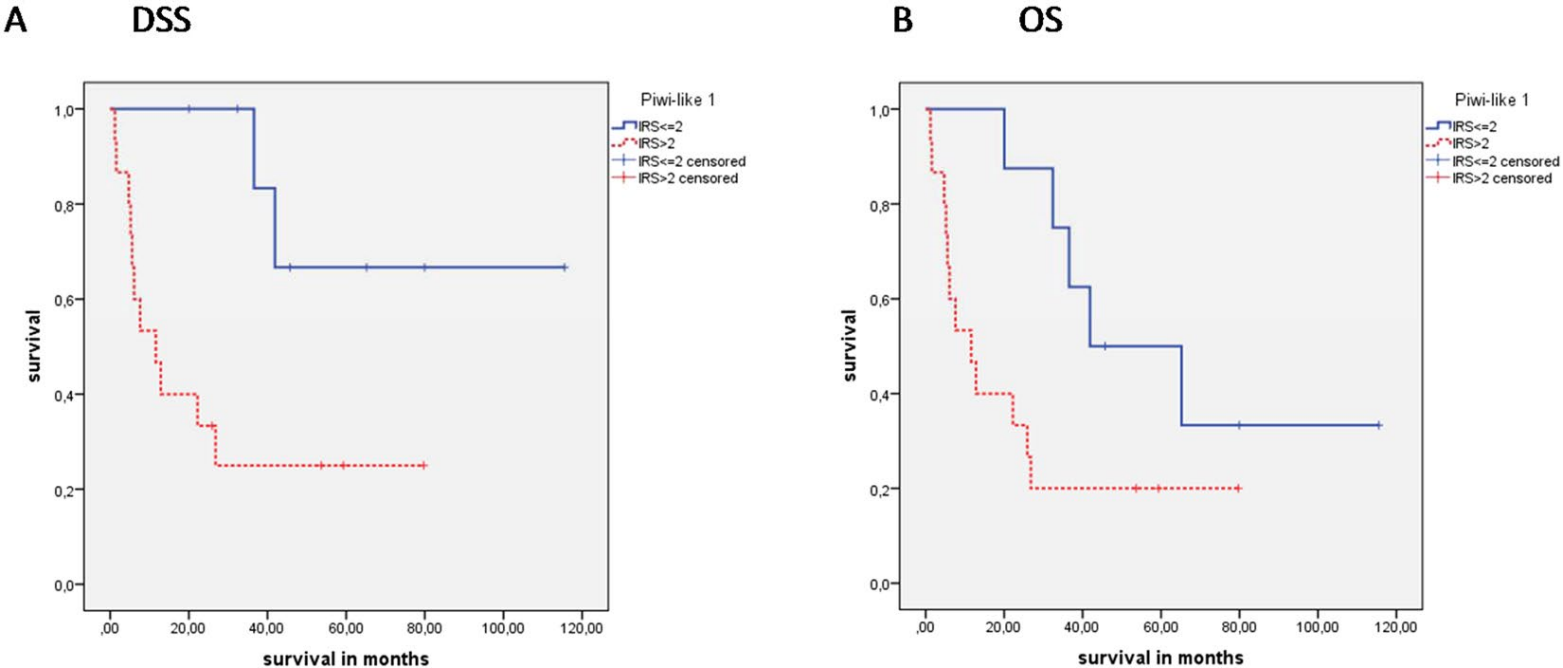
Kaplan-Meier analyses: Association of Piwi-like 1/-2 staining with prognosis in GATA3+/CK5− patients (luminal subtype). Piwi-like 1 protein expression was associated with (**A**) DSS (P = 0.014), (**B**) OS (P = 0.049; all log rank tests, Kaplan-Meier analyses).

In addition, we tested whether Piwi-like 1/-2 staining was associated with OS, DSS or RFS in the luminal type (GATA3+/CK5− group). Piwi-like 1 staining was significantly associated with OS and DSS but not with RFS. Patients with Piwi-like 1 positivity had an overall survival of 24.7 months and a tumor-specific survival of 27.4 months, whereas those with negative Piwi-like 1 staining had an overall survival of 65.7 months and a tumor-specific survival of 90.1 months (P = 0.049 and P = 0.014; Table 2; Fig. 5).

Piwi-like 1 expression was not significantly associated with OS in univariate or multivariate Cox’s regression analysis. For DSS, univariate Cox’s regression analysis revealed that compared with Piwi-like 1 negativity, Piwi-like 1 positivity had a 5.1-fold risk for tumor-specific death (P = 0.028; Table 3). However, multivariate Cox’s regression analysis (adjusted for tumor grade and tumor stage) did not show a significantly increased risk for DSS (RR = 4.7; P = 0.068).

Piwi-like 2 staining was not associated with OS, DSS and RFS in the GATA3+/CK5− group.

## Discussion

In this study, protein expression of Piwi-like 1 and Piwi-like 2 was analyzed in tumors from 95 MIBC patients and they were associated with clinico-pathological and survival data. Expression of both proteins was positively correlated with lymph node metastasis, CK20 staining, and GATA 3 staining; moreover, the expression levels of both Piwi-like proteins were correlated with each other. In addition, a negative correlation was detected with disease-specific survival, recurrence, Ki67/MIB1 staining, and CK5 staining. Our data support previous findings of a correlation between Piwi-like 1 and/or 2 staining with lymph node metastases in gastric, ovarian, breast and colorectal cancer^30–34^. A correlation of Piwi-like 1/-2 expression with GATA3, CK20 or CK5 has not been reported yet. However, a correlation between the expression of both Piwi-like proteins could be expected as they show on the protein level 34% sequence homology^35^. We could show that transcript levels of Piwi-like 1 and -2 were significantly correlated in renal cell carcinoma^36^, but a reciprocal regulation of Piwi-like 1 and Piwi-like 2 at the RNA level has been suggested in colorectal cancer^37^. We detected a negative correlation between Piwi-like 1/-2 protein expression and Ki67 protein expression. This is somewhat in contrast with the findings that Piwi-like 2 protein expression in the nucleus was significantly correlated to Ki67 expression in breast cancer^38^, and cytoplasmic expression of Piwi-like 1 protein was associated with Ki67 expression in human gastric cancer cells^39^ and in gliomas^40^. There might be tumor cell-specific differences in the expression of Piwi-like proteins but also differences in the number of active proliferating cells with Ki67 expression between the tumor entities.

We also showed for the first time that positive Piwi-like 1 protein (IRS > 2) detection was significantly associated with poor DSS and that in multivariate Cox’s analysis (adjusted to tumor stage and tumor grade), Piwi-like 1 positivity appeared as an independent prognostic factor for DSS in MIBC. This is in line with our previous findings and those of others, showing that positive cytoplasmic expression of Piwi-like 1 (HIWI) protein was significantly associated with poorer DSS in esophageal squamous cell carcinoma^41^, colorectal cancer^42^ and in pancreatic carcinomas^43^. In addition, Piwi-like 2 positivity (IRS > 2) was significantly associated with DSS and RFS, and in multivariate Cox’s analysis (adjusted to tumor stage and tumor grade), Piwi-like 2 positivity appeared as an independent prognostic factor for DSS and RFS in MIBC. This finding is noticeable since Piwi-like 2 positivity is considered in other tumor entities to be a predictor for OS only^17–19^, but in colon cancer, Piwi-like 2 positivity was associated with poorer five-year metastasis-free survival^44^.

In our previous study of chemotherapy-treated bladder cancer, patients were investigated with a different Piwi-like 2 antibody, a weak cytoplasmic staining pattern (IRS 1–2) was associated with poor DSS and tumor progression^28^. The group of patients with negative Piwi-like 2 staining (IRS = 0) showed in this and in the previous study a rather good prognosis. However, in the previous study, patients with moderate or strong Piwi-like 2 staining (IRS 3–4 and IRS 6–12) showed a better DSS and progression-free survival, whereas in this study, patients with positive staining (IRS > 2) had a poorer DSS and RFS. The reason for this difference could be that in the previous study all 202 patients were treated with chemotherapy, whereas in this study, among a group of 95 patients, only 27 (28%) received chemotherapy.

How could Piwi-like 2 expressed in the cytoplasm affect DSS, RFS and chemotherapy response? In protozoa, i.e., Leishmania species, a PIWI-like protein homolog is localized in the cytoplasm as a regulator of RNA stability and translation^45^, suggesting an ancient role of Piwi-like proteins. It has been shown that human Piwi-like 2 binds to keratin 8 and p38 MAPK through its PIWI domain and forms a Piwil2/K8/P38 triple protein-protein complex. In this way, it represses p53 phosphorylation through p38 MAPK, which is necessary for P53-induced apoptosis, and by its binding to keratin 8 it protects cells from Fas-mediated apoptosis^46^. Furthermore, Piwi-like 2 can form with STAT3 and c-Src triple protein-protein complexes, and phosphorylated STAT3 will then translocate to the nucleus, where it binds to the P53 promoter and represses P53 transcription^47^. In addition, overexpression of Piwi-like 2 was found to contribute to cisplatin resistance in human ovarian cancer cell lines, suggesting that Piwi-like 2 could be a marker for cisplatin resistance in cancer chemotherapy^26^. Vice versa, knockdown of Piwi-like 2 expression in these cell lines resulted in their enhanced sensitivity to cisplatin and decreased their efficiency for removing cisplatin-induced DNA intra-strand crosslinks^26^. Altogether, Piwi-like 2 can inhibit apoptosis, which may affect prognosis and therapy responses. However, the role of Piwi-like 2, especially in the cytoplasm, certainly needs further investigation.

Most interestingly, we could identify three groups where Piwi-like 2 could be used to separate patients with a poorer and a better prognosis. At first, in the patients with tumors with low-proliferation (Ki67 ≤ 30%) but not in patients with high-proliferating tumors (Ki67 > 30%), Piwi-like 2 positivity was associated with a poorer DSS and OS. Second, Piwi-like 2 positivity was associated with a poorer DSS, OS and RFS in patients with tumor cells that are CK5-positive/GATA3-negative but not in those with GATA3-positive/CK5-negative tumor cells. CK5-positive/GATA3-negative cells are characteristic of the so-called basal cell type of bladder cancer that can be identified consistently in several subtyping approaches for bladder cancer^4,6,7^. Recently, it was shown that the basal cell type is the type that responds best to chemotherapy in bladder cancer^6–8^. Third, in patients of the squamous differentiated subtype of BCa but not in the non-squamous differentiated subtype of BCa. Interestingly, most of the squamous differentiated subtype of BCa belong to the basal cell type^6^. Piwi-like 2 positivity was associated with shorter OS, DSS and RFS in patients of the squamous differentiated subtype of BCa and appeared as independent prognostic marker in this subtype. However, Piwi-like 1 positivity appeared also as independent prognostic marker in the squamous differentiated subtype of BCa. In addition, in tumors with GATA3-positive/CK5-negative cells, considered as luminal type, Piwi-like 1 positivity was associated with shorter OS and DSS. But Piwi-like 1 was not an independent prognostic factor in the GATA3-positive/CK5-negative group.

Although, we have no primary data for a correlation between Piwi-like1/-2 expression and chemotherapy response, there are reports that show a relationship. Wang *et al*. describe that Piwi-like 2 level was enhanced in cisplatin-resistant ovarian cancer cell lines^26^. Furthermore, a report shows that chemotherapy response has an U-shape for the association of Piwi-like 1 (HIWI) protein expression and OS. A moderate level (but not low or strong levels) of Piwi-like 1 protein could be associated with an increased risk of death and poor chemotherapy response in epithelial ovarian cancer patients^48^.

Finally, results from the literature, showing that Piwi-like 1 protein affects DNA methylation and Piwi-like 2 protein histone acetylation^22,23,25^, may support the hypothesis that patients with Piwi-like positive BCa of the squamous differentiated subtype may respond to DNA methylase transferase inhibitors or histone deacetylase inhibitors.

Shortcomings of our study are the limited sample and subgroup size analyzed, the retrospective approach and that immunohistochemical analysis is not an objective measurement of protein levels.

In summary, Piwi-like 1 and -2 positivity are associated with clinico-pathological factors and survival. Therefore, both Piwi-like proteins are suggested as prognostic biomarkers for MIBC patients.

## Material and Methods

### Patients and tumor material

Tissue microarrays (TMA) with formalin-fixed and paraffin embedded tumor samples of 95 MIBC patients were investigated in this study. The TMA was prepared as follows: HE slides were scanned (Panoramic P250, 3DHistech, Budapest, Hungary) and annotated using a TMA annotation tool (Caseviewer v2). Four cores (diameter 1 mm; two cores from the invasion margin, two cores from the tumor center) were taken utilizing an automated tissue microarrayer (TMA Grandmaster, 3DHistech, Budapest, Hungary) as described previously^49,50^. The research carried out on human subjects is in compliance with the Helsinki Declaration. All patients gave written informed consent. The study is based on the approvals of the Ethic Commission of the University Hospital Erlangen (No. 3755 and No. 329_16B). Tumor histology was reviewed by two uropathologists (AH, ME). An overview of the clinico-pathologic parameters of the patients included in this study is given in Table 1.

### Immunohistochemistry

For the study of Piwi-like 1 and Piwi-like 2 protein expression, a manual IHC protocol was applied as previously described^28^. Briefly, after heat pretreatment at 120 °C for 5 min with TE–buffer pH 9 and peroxidase blocking (Dako, Hamburg, Germany), primary antibodies against Piwi-like 1 (polyclonal goat IgG, N-17; Cat.-No. sc22685; dilution 1:50; Santa Cruz, Heidelberg, Germany) and Piwi-like 2 (polyclonal goat IgG, K-18; Cat.-No. sc67502; dilution 1:50; Santa Cruz) were applied for 30 min. After incubation with a respective HRP-labeled secondary antibody polymer (Anti-Goat- Histofine Nichirei, Medac, Wedel, Germany) for 30 min, a DAB1 substrate chromogen solution (Dako) was added for 10 min. The slides were counterstained for 1 min with hematoxylin (Merck, Darmstadt, Germany). Between all of the steps, the slides were washed with buffer from Dako and all of the incubation steps were performed at room temperature. IHC staining of CK5 (monoclonal mouse IgG, clone XM26; dilution 1:50; Diagnostic BioSystems, Pleasanton, USA), CK20 (monoclonal mouse IgG, clone Ks 20.8; dilution 1:25; Dako, Glostrup, Denmark), GATA3 (monoclonal mouse IgG, clone L50-823; dilution 1:100; Sigma-Aldrich, Taufkirchen, Germany) and Ki67 (monoclonal mouse IgG, clone M7240; dilution 1:75; Dako) were performed on a fully automated Ventana Benchmark Ultra autostainer (Ventana, Tucson, Arizona, USA). Sections were deparaffinized and antigens retrieved by heating the sections in a pH 8.4 Tris/borate/EDTA solution (Ventana). Endogenous peroxidase was blocked with 1% H_2_O_2_. Visualization of bound antibody was performed using the ultraVIEW TM DAB system (Ventana). All sections were counterstained with hematoxylin II/Mayer’s hematoxylin (Ventana).

Stained specimens were viewed at an objective magnification of ×100 and ×200. Expression of Piwi-like 1 and Piwi-like 2 was detected in the cytoplasm by assessing the percentage of stained tumor cells and the staining intensity semi-quantitatively. The percentage of positive cells was scored as follows: 1, 1–9% positive cells; 2, 10–50%; 3, 51–80%; and 4, >80% positive cells. Staining intensity was scored as 0, negative; 1, weak; 2, moderate; and 3, strong. The immunoreactive score (IRS) was calculated as the product of staining percentage and staining intensity, resulting in an IRS from 0 to 12^51^. Negative control slides without the addition of primary antibody were included for each staining experiment. From each sample a core from the center and a core from the invasive front were analyzed. Afterwards, the average of both IRS scores was determined. For survival analysis, patients were grouped as Piwi-like 1/-2 negative (IRS ≤ 2) and Piwi-like1/-2 positive (IRS > 2) as an IRS of 2 can be applied to distinguish between IRS negative and IRS positive patients^52^. For characterization of basal and luminal type of BCa, protein expression of cytokeratin 5 (CK5) and GATA binding protein 3 (GATA3) was assessed by IHC. The CK5+ (IRS > 2) and GATA3− (IRS ≤ 2) tumors were considered as basal type and the GATA3 + (IRS > 2) and CK5− (IRS ≤ 2) were counted as luminal type. Photos were taken with a Leica DM 4000B microscope with 20x HC PL Fluotar objective (Leica, Wetzlar, Germany) and with a Jenoptik Gryphax Arktur camera (Jenoptik AG, Jena, Germany).

### Statistical analyses

The associations between the IHC and clinical data were calculated using the Chi^2^-test or the Mann-Whitney test. The associations of the expression of Piwi-like 1/-2 with overall survival (OS) or disease-specific survival (DSS) were determined in univariate (Kaplan-Meier analysis and Cox’s regression hazard models) and multivariate analyses (Cox’s regression hazard models, adjusted for tumor grade and tumor stage). A p-value of less than 0.05 was considered statistically significant. Statistical analyses were performed with the SPSS 21.0 software package (SPSS Inc., Chicago, IL).

## Electronic supplementary material

Supplementary Table
