# Supplementary material for: Piwi-like 1 and -2 protein expression levels are prognostic factors for muscle invasive urothelial bladder cancer patients
Source: Sci Rep. 2018 Dec 6;8:17693. doi: 10.1038/s41598-018-35637-4 (PMC6283838; doi:10.1038/s41598-018-35637-4)
Supplement: Supplementary file 1 — Supplementary Table [file 41598_2018_35637_MOESM1_ESM.docx]

**Supplementary Table**

Piwi-like 1 and -2 protein expression levels are prognostic factors

for muscle invasive urothelial bladder cancer patients

Markus Eckstein^1^, Rudolf Jung^1^, Katrin Weigelt^2^, Danijel Sikic^2^, Robert Stöhr^1^, Carol Geppert^1^, Abbas Agaimy^1^, Verena Lieb^2^, Arndt Hartmann^1^, Bernd Wullich^2^, Sven Wach^2^, Helge Taubert^2^*

^1^Institute of Pathology, University Hospital Erlangen, FAU Erlangen-Nürnberg, Germany

^2^Department of Urology and Pediatric Urology, University Hospital Erlangen, FAU Erlangen-Nürnberg, Germany

**Suppl. Tab. Immunoreactive score (IRS) for Piwi-like 1/-2 protein expression in MIBC**

| **IRS** | **Piwi-like 1** | **Piwi-like 2** |
| --- | --- | --- |
| 0 | 22 | 12 |
| 1 | 17 | 16 |
| 2 | 19 | 22 |
| 3 | 18 | 20 |
| 4 | 7 | 15 |
| 5 | 6 | 6 |
| 6 | 4 | 3 |
| 7 | 1 | 1 |
| 9-12 | 0 | 0 |
|  |  |  |
| IRS≤2 | 58 | 50 |
| IRS>2 | 37 | 45 |
